# Using the Health Belief Model to Predict Vaccination Intention Among COVID-19 Unvaccinated People in Thai Communities

**DOI:** 10.3389/fmed.2022.890503

**Published:** 2022-06-03

**Authors:** Katekaew Seangpraw, Tharadon Pothisa, Sorawit Boonyathee, Parichat Ong-Artborirak, Prakasit Tonchoy, Supakan Kantow, Nisarat Auttama, Monchanok Choowanthanapakorn

**Affiliations:** ^1^School of Public Health, University of Phayao, Phayao, Thailand; ^2^School of Medicine, University of Phayao, Phayao, Thailand; ^3^Faculty of Public Health, Chiang Mai University, Chiang Mai, Thailand

**Keywords:** Health Belief Model, intention, COVID-19 vaccine, Thai people, community

## Abstract

**Background:**

The coronavirus disease 2019 (COVID-19) pandemic has become one of the biggest challenges to individual health and the public health system worldwide. COVID-19 morbidity and mortality are increasing, impacting almost every country including Thailand. This study used the Health Belief Model (HBM) as a framework to examine the intention of unvaccinated people living in northern Thailand to receive COVID-19 vaccines.

**Methods:**

This cross-sectional study was conducted during October and November 2021. A total of 1,024 participants who are currently living in four northern provinces of Thailand, Chiang Mai, Chiang Rai, Lamphun, and Phayao, were recruited to participate in the study. The questionnaire was developed using an HBM structure to obtain information about the perceived severity, perceived susceptibility, self-efficacy, perceived benefits and barriers, cues to action, and preventive behaviors relating to COVID-19 vaccination and the decision to become vaccinated. Multiple linear regression was used to analyze the data.

**Results:**

The unvaccinated participants were an average of 44.45 ± 16.63 years of age and more than half were women (54.5%). The COVID-19 preventive behavior score used perceived severity (B = 0.26), self-efficacy (B = 0.51), perceived benefits and barriers (B = 0.11), and cues to action (B = 0.18) after adjusting for age, underlying disease, and body mass index (*R*^2^ = 42.5%). The COVID-19 vaccination decision score was positively correlated with perceived severity (B = 0.13), perceived susceptibility (B = 0.25), perceived benefits and barriers (B = 0.21), and cues to action (B = 0.27) after adjusting for underlying disease (*R*^2^ = 38.7%).

**Discussion:**

The results demonstrated the usefulness of using the HBM structure to understand individual intention to receive a COVID-19 vaccine. Communities should consider a COVID-19 health campaign and programs that use the HBM model as a framework for altering perceptions and beliefs about the COVID-19 vaccine and improving vaccination rates among unvaccinated people in rural northern Thailand.

## Introduction

Coronavirus disease 2019 (COVID-19) is an infectious disease caused by the SARS-CoV-2 virus that is responsible for respiratory problems ranging from mild to severe (1). This virus has had a devastating impact on healthcare systems worldwide and in all areas of human life, infecting a large number of people and resulting in millions of deaths (1, 2). The World Health Organization (WHO) raised the level of COVID-19 to a global pandemic and this disease has impacted the entire world, including Thailand (1, 3). Thailand declared COVID-19 a dangerous communicable disease under the Communicable Diseases Act B.E. 2558 (3, 4). According to data from the Department of Disease Control, Ministry of Public Health, Thailand as of January 31, 2022, there have been 2,425,412 confirmed cases of COVID-19, and 22,173 deaths, representing a mortality rate of 0.91% (4). While many treatments and vaccines are currently being studied, there is no absolute cure for COVID-19 (5, 6). As a result, governments in Thailand and around the world have enforced laws and regulations including border closures, travel bans, state quarantines, and community involvement to prevent viral spread (5–7).

Distributing COVID-19 vaccines is currently the most important public health pandemic mitigation measure (2). The success of the COVID-19 vaccine program depends on the number of distributed vaccines and the immunization rate of the population (2). The Emergency Use Authorization (EUA) recently approved the use of vaccines in several countries including Thailand (8, 9). According to health research, there has been increasing “vaccination hesitancy” within the general population over the past decade, and this increased significantly with regard to COVID-19 vaccination (8, 10). Vaccination hesitancy includes refusal, uncertainty, and delay in accepting the vaccination even when vaccines are available (4, 10–13). It is very important to understand the predictors and determinants that influence the intention to receive COVID-19 vaccines (12, 13). This is critical to the design of public health programs that optimize vaccination rates when vaccines are available for all populations (14, 15).

In total, 59.6% of Thai people received the COVID-19 vaccine from 28 February 2020 to 1 December 2021 (9, 16). Since December 10, 2021, 42,405,356 people have received at least two vaccine doses, representing 58.9% of the total population (9). However, this proportion is not enough to reduce the rate of COVID-19 infection in the Thai population (9). The current vaccines used in Thailand are fully registered and approved for emergency use. They are divided into two groups: (1) vaccines provided by the Department of Disease Control at the Ministry of Public Health including Sinovac, AstraZeneca, and Pfizer, and (2) alternative vaccines from Sinopharm and Moderna (9). There are some side effects associated with the vaccine, including pain, swelling at the injection site, fatigue, headache, fever, muscle and joint aches, nausea, dizziness, low blood pressure, rapid heartbeat, shortness of breath, nasal congestion, and thromboembolism (3, 9). When the third wave of the COVID-19 pandemic occurred in April 2021, individuals became more interested in getting vaccinated (9). However, factors influencing vaccination hesitancy, including uncertainty about vaccine safety, benefits, the risk of acquiring COVID-19 infection after vaccination, and the risk associated with receiving government issues vaccines are important issues to address within the population (3, 9). Studies in the United States (US) and elsewhere found that individual willingness to receive the COVID-19 vaccine was initially very low (14, 15). This was improved by studies showing that COVID-19 vaccination helps to reduce the rate of infection, hospitalization, and mortality (17). However, even when vaccines are fully available, it is difficult for governments to encourage unvaccinated populations to trust and receive them. This is a particular challenge for the Thai government.

The Health Beliefs Model (HBM) is one of the most widely used theories for understanding health behaviors among people (18, 19). This theory consists of several key structures including perceived severity, perceived susceptibility, self-efficacy, perceived benefits and barriers, and cues to action (20). The HBM structure is recognized as an important predictor of influenza vaccination exposure, and its structure differs depending on the national context and epidemiological conditions (21, 22). In this study, the HBM was used as an analytical framework to examine individual perceptions about and willingness of unvaccinated people living in northern Thailand to receive COVID-19 vaccines. The influence of various factors on the intention to receive vaccination was also assessed. Understanding how the HBM structure influences COVID-19 vaccination could help in the development of tailored activities to increase vaccine acceptance and reduce hesitancy in rural areas of Thailand.

## Materials and Methods

This was a cross-sectional study included under the Unit of Excellence Project. It is an ongoing survey of vaccination among residents of northern Thailand. Data collection was performed during October and November 2021 in the rural communities of four upper northern provinces of Thailand, Chiang Mai, Chiang Rai, Lamphun, and Phayao, where there were high rates of COVID-19 infection and outbreaks. The geographical research area is in a remote and hard to access region located 30–40 km from the city center. The sampling technique and participant recruitment were described previously (23). In brief, one district was chosen from each province, and one sub-district of each district was obtained by random sampling. Participants were randomly selected from each sub-district. All unvaccinated participants who took part in our earlier study (23) were invited to participate. In total, 1,024 of 1,524 subjects from four provinces were enrolled. The participants, females and males ≥20 years of age who were living in the research area for at least 1 year, were considered eligible for the study. All participants signed a written consent form to participate in the study prior to data collection.

The researcher set up a meeting to train 15 research assistants who were able to communicate in the local language from each province. The researcher then translated the official language into the local northern language. This ensured that everyone would understand the questions and be able to communicate effectively with the research staff and study participants. The researcher contacted the health officers from each provincial health office asking permission to conduct research in those areas. However, because the research was performed during the third wave of COVID-19 outbreaks in Thailand, the research team had to follow government measures announced in each community area. The participants were interviewed by a research assistant using face-to-face questionnaires.

The questionnaire used an HBM structure for quantitative data collection consisting of six parts which were obtained from a prior study and developed to be more suitable for the community context of this project (18, 19, 24, 25): (1) perceived severity of COVID-19 infection consisting of 13 items (for example, COVID-19 causes death; the main symptoms of COVID-19 infection are fever, fatigue, dry cough, and body aches), (2) perceived susceptibility of COVID-19 infection consisting of 12 items (for example, eating and touching wild animals and cohabiting in crowded places such as markets, communities may result in getting infected with COVID-19), (3) self-efficacy toward COVID-19 consisting of 12 items (for example, I cover my nose with a tissue every time when coughing or sneezing; I will immediately put on a mask when I am in a crowded place), (4) perceived benefits of and barriers to COVID-19 prevention, consisting of 10 items (For example, vaccination against COVID-19 can help prevent the infection; it is annoying to have body temperature check all the time when enter a public place), (5) cues to action toward the COVID-19 vaccine consisting of 12 items (For example, you are worried about the short-term side effects of the COVID-19 vaccine; you are afraid of the injection), and (6) decision making about receiving the COVID-19 vaccine consisting of 10 items (for example, to help protect yourself and others, are you mentally prepared to get the COVID-19 vaccine that can possibly give you short-term side effects such as abdominal pain or nausea).

Health Belief Model items were rated on the Likert scale (Ranging: 1–3 from “disagree” to “agree”). For each part, the scores of each item were totaled. The scores were divided into three categories: High level = ≥80%, Moderate level = 60–79%, and Low level = <60%. Our previously published socio-demographic and COVID-19 preventive behavior data on the study populations was used (23). The behavior questions were designed to be suitable for participants living in rural areas (26, 27) and consisted of 15 items with three responses: never, sometimes or ≤4 times/week, and regularly or ≥5 times/week (for example, you dispose your mask after you use it in sealed plastic bag or covered bin; you wash your hands with soap after coughing, sneezing, or blowing your nose). Each of the research tools passed a content validity check by three experts in the fields of internal medicine, behavioral health, and public health. The questionnaire reliability was tested on 30 participants. Parts 1–6 obtained a Cronbach's alpha coefficient of 0.76, 0.80, 0.77, 0.79, 0.82, and 0.78, respectively.

### Statistical Analysis

Data analysis was performed using SPSS Version 17 software (SPSS Inc., Chicago, IL, United States), licensed from Chiang Mai University. Descriptive statistics on the population used frequency (*n*), percentage (%), arithmetic mean, standard deviation (SD), minimum (min), and maximum (max). The unpaired *t*-test and one-way ANOVA were used to compare the differences in the vaccination decision score between two groups (sex, marital status, perceived financial status, underlying disease, smoking, and alcohol consumption), and more than two groups (age, education, occupation, and BMI), respectively. The Welch's test was used when equal variances were not assumed. The Pearson correlation coefficient (*r*) was used to examine the correlation between the variables, perceived severity, perceived susceptibility, self-efficacy, perceived benefits and barriers, cues to action, COVID-19 vaccination decision, and preventive behaviors. The HBM variables used to predict COVID-19 preventive behaviors and vaccination decision were examined using multiple linear regression. The demographic variables including age, underlying disease, and BMI were controlled because they are important factors that influence preventive behaviors (23), while underlying disease was included in the model to predict COVID-19 vaccination decision. Tolerance and variance inflation factor (VIF) were measured to check multicollinearity in the regression model. The level of statistical significance was set at 0.05.

## Results

### Participant Characteristics

Of the 1,024 participants, 54.5% were female (Table 1). The mean age of the respondents was 44.45 years (SD = 16.63). Nearly half were married, and the remaining were single/divorced (49.0 and 51.0%, respectively). Most participants were educated through primary and secondary school (31.8 and 31.0%, respectively). The most common occupations were general employee, followed by merchant/self-employed, and farmer (29.5, 17.2, and 15.3%, respectively). More than half of the participants had a sufficient income status (56.3%). Nearly half had a normal BMI (49.7%) and 71.6% had no underlying disease diagnosed by a doctor. Most participants did not drink alcohol (61.7%) or smoke (57.9%). Underlying disease was found to be statistically related to the decision to vaccinate (*P* < 0.001, Table 1).

**Table 1:** General characteristics of participants (*n* = 1,024).

**Variable**	***n* (%)**	**Vaccination decision (score)**	
		**Mean ±SD**	***P*-value**
**Sex**			
Male	466 (45.5%)	20.90 ± 3.68	0.716^a^
Female	558 (54.5%)	20.98 ± 3.62	
**Age (years)**			
<30	267 (26.1%)	20.93 ± 3.96	0.786^b^
30–39	201 (19.6%)	20.73 ± 3.57	
40–49	137 (13.4%)	20.81 ± 3.30	
50–59	181 (17.7%)	21.15 ± 3.77	
≥60	238 (23.2%)	21.05 ± 3.44	
Mean ± SD	44.45 ± 16.63		
Min.—Max.	18–89		
**Marital status**			
Single/Widowed/ Divorced/Separate	522 (51.0%)	20.97 ± 3.66	0.836^a^
Married	502 (49.0%)	20.92 ± 3.64	
**Education**			
No	170 (16.6%)	21.15 ± 3.71	0.805^c^
Primary school	326 (31.8%)	20.91 ± 3.57	
Secondary school	317 (31.0%)	20.82 ± 3.70	
Diploma/Bachelor degree	211 (20.6%)	21.01 ± 3.64	
**Occupation**			
No	162 (15.8%)	20.81 ± 3.73	0.381^c^
Government/Private sector	84 (8.2%)	21.33 ± 4.04	
Farmer	157 (15.3%)	21.34 ± 3.51	
General employee	302 (29.5%)	20.94 ± 3.65	
Merchant/Self-employed	176 (17.2%)	20.53 ± 3.50	
Student	143 (14.0%)	20.93 ± 3.60	
**Perceived financial status**			
Insufficient, having financial difficulties	447 (43.7%)	20.95 ± 3.55	0.948^a^
Sufficient, with and without savings	577 (56.3%)	20.94 ± 3.72	
**BMI**			
<18.5 kg/m^2^	61 (6.0%)	21.02 ± 3.47	0.711^c^
18.5–22.9 kg/m^2^	509 (49.7%)	20.84 ± 3.76	
23.0–24.9 kg/m^2^	232 (22.7%)	21.18 ± 3.60	
≥25.0 kg/m^2^	222 (21.7%	20.90 ± 3.49	
**Underlying disease**			
No	733 (71.6%)	21.19 ± 3.72	<0.001^a^
Yes	291 (28.4%)	20.33 ± 3.38	
**Smoking**			
No	632 (61.7%)	20.82 ± 3.70	0.183^a^
Yes	392 (38.3%)	21.14 ± 3.55	
**Alcohol drinking**			
No	593 (57.9%)	20.95 ± 3.76	0.914^a^
Yes	431 (42.1%)	20.93 ± 3.48	

*^a^Independent T-Test*.

*^b^Welch's ANOVA*.

*^c^One-way ANOVA*.

### HBM Constructs Among Unvaccinated Participants

Table 2 shows the results of the HBM questions. Over half of all respondents had a moderate perceived severity score (65.4%, mean = 25.88, SD = 3.56) and a moderate perceived susceptibility score (56.0%, mean = 24.25, SD = 3.67). Nearly half of the respondents had moderate and high self-efficacy (48.1 and 32.1%, respectively, mean = 25.36, SD = 3.94), while 76.9% had a moderate level of perceived benefits and barriers (mean = 20.44, SD = 2.50). Interestingly, 50.3% of respondents had a moderate cue to action and decided to accept the COVID-19 vaccine at moderate and high levels (53.2, 26.4%, mean = 20.94, SD = 3.64). Finally, 39.9 and 35.4% of respondents had moderate and high scores, respectively, for COVID-19 preventive behaviors (mean = 32.50, SD = 4.97).

**Table 2:** Descriptive of the Health Belief Model constructs and vaccination decision (*n* = 1,024).

**Variables**	***n***	**%**
**Perceived severity of COVID-19 infection**		
Low level (13–22 scores)	255	24.9
Moderate level (23–30 scores)	670	65.4
High level (31–39 scores)	99	9.7
Mean ± SD	25.88 ± 3.56	
Min.—Max.	19–39	
**Perceived susceptibility of COVID-19 infection**		
Low level (12–20 scores)	229	22.4
Moderate level (21–27 scores)	574	56.0
High level (28–36 scores)	221	21.6
Mean ± SD	24.25 ± 3.67	
Min.—Max.	17–34	
**Self-efficacy toward COVID-19**		
Low level (12–20 scores)	203	19.8
Moderate level (21–27 scores)	492	48.1
High level (28–36 scores)	329	32.1
Mean ± SD	25.36 ± 3.94	
Min.—Max.	18–35	
**Perceived benefits and barriers to COVID-19 prevention**		
Low level (10–17 scores)	127	12.4
Moderate level (18–23 scores)	787	76.9
High level (24–30 scores)	110	10.7
Mean ± SD	20.44 ± 2.50	
Min.—Max.	14–30	
**Cues to action toward the COVID-19 vaccine**		
Low level (12–20 scores)	228	22.3
Moderate level (21–27 scores)	515	50.3
High level (28–36 scores)	281	27.4
Mean ± SD	24.60 ± 4.28	
Min.—Max.	16–35	
**COVID-19 preventive behaviors**		
Low level (15–27 scores)	253	24.7
Moderate level (28–35 scores)	409	39.9
High level (36–45 scores)	362	35.4
Mean ± SD	32.50 ± 4.97	
Min.—Max.	24–43	
**COVID-19 vaccination decision**		
Low level (10–17 scores)	209	20.4
Moderate level (18–23 scores)	545	53.2
High level (24–30 scores)	270	26.4
Mean ± SD	20.94 ± 3.64	
Min.—Max.	14–29	

### Using the HBM to Predict COVID-19 Preventive Behaviors and Vaccination Decision Among Unvaccinated Participants

Correlations between all variables in the study, including the HBM constructs, COVID-19 preventive behaviors, and vaccination decision, were significant at the 0.01 level (Table 3). As a result of the high positive correlation between perceived susceptibility and self-efficacy (*r* = 0.651), only one variable was included in the regression model to minimize collinearity. Linear regression analysis showed that perceived severity, self-efficacy, perceived benefits and barriers, and cues to action predicted COVID-19 preventive behaviors after controlling for age, underlying disease, and BMI (*R*^2^ = 42.5%), with self-efficacy being the strongest predictor (B = 0.505; Table 4). Perceived severity, perceived susceptibility, perceived benefits and barriers, cues to action, and underlying disease accounted for 38.7% of the variation in COVID-19 vaccination decision, with cues to action being the strongest predictor (B = 0.267; Table 5).

**Table 3:** Correlation coefficients (*r*) between the Health Belief Model constructs, COVID-19 preventive behaviors, and vaccination decision (*n* = 1,024).

**Variable**	**1**	**2**	**3**	**4**	**5**	**6**	**7**
1. Perceived severity	1						
2. Perceived susceptibility	0.369**	1					
3. Self-efficacy	0.395**	0.651**	1				
4. Perceived benefits and barriers	0.221**	0.338**	0.308**	1			
5. Cues to action	0.370**	0.448**	0.538**	0.416**	1		
6. Preventive behaviors	0.415**	0.504**	0.585**	0.282**	0.473**	1	
7. Vaccination decision	0.365**	0.484**	0.352**	0.383**	0.531**	0.343**	1

^**^Significance at the 0.01 level (two-tailed).

**Table 4:** Association between the Health Belief Model constructs and COVID-19 preventive behavior score by multiple linear regression.

**Factor**	**B**	**S.E**.	**Beta**	***P*-Value**	**95% CI**	**Tolerance**	**VIF**
Constant	5.752	1.289		<0.001	3.222, 8.283		
**Age**
<30 years	Ref.						
30–39 years	−0.060	0.357	−0.005	0.867	−0.759, 0.640	0.701	1.426
40–49 years	0.284	0.402	0.019	0.480	−0.505, 1.073	0.750	1.333
50–59 years	1.340	0.379	0.103	<0.001	0.597, 2.084	0.672	1.487
≥60 years	0.973	0.363	0.083	0.007	0.261, 1.685	0.598	1.671
Underlying disease (yes)	−0.763	0.296	−0.069	0.010	−1.344, −0.182	0.789	1.267
**BMI**
<18.5 kg/m^2^	0.701	0.517	0.033	0.176	−0.314, 1.715	0.940	1.064
18.5–22.9 kg/m^2^	Ref.						
23.0–24.9 kg/m^2^	−0.209	0.303	−0.018	0.492	−0.803, 0.386	0.874	1.145
≥25.0 kg/m^2^	0.813	0.312	0.067	0.009	0.200, 1.425	0.850	1.177
Perceived severity	0.262	0.038	0.187	<0.001	0.187, 0.337	0.755	1.325
Self-efficacy	0.505	0.037	0.400	<0.001	0.432, 0.578	0.655	1.528
Perceived benefits and barriers	0.110	0.053	0.055	0.038	0.006, 0.214	0.804	1.243
Cues to action	0.181	0.035	0.156	<0.001	0.112, 0.251	0.614	1.628

*B, unstandardized coefficients; S.E, standard error; Beta, standardized coefficients; 95% CI, 95% confidence interval; VIF, variance inflation factor; Ref, reference group*.

**Table 5:** Association between the Health Belief Model constructs and COVID-19 vaccination decision score by multiple linear regression.

**Factor**	**B**	**S.E**.	**Beta**	***P*-value**	**95% CI**	**Tolerance**	**VIF**
Constant	0.670	0.957		0.484	−1.208, 2.548		
Underlying disease (yes)	0.130	0.206	0.016	0.529	−0.274, 0.533	0.928	1.077
Perceived severity	0.133	0.029	0.129	<0.001	0.077, 0.189	0.773	1.294
Perceived susceptibility	0.249	0.029	0.250	<0.001	0.193, 0.305	0.728	1.373
Perceived benefits and barriers	0.205	0.040	0.141	<0.001	0.127, 0.284	0.796	1.257
Cues to action	0.267	0.025	0.314	<0.001	0.218, 0.317	0.684	1.461

## Discussion

The Health Belief Model (HBM) is a conceptual framework that is used to describe, predict, and influence the health behavior of individuals or groups (19, 20). This study showed significant positive correlations between perceived severity, perceived susceptibility, self-efficacy, perceived benefits and barriers, cues to action, preventive behaviors, and vaccination decision among COVID-19 unvaccinated people living in rural areas of northern Thailand. The structural perception of HBM can explain 38.7% of the variation in vaccination decision scores. Adoption of the HBM structure as a conceptual framework helped to predict vaccine decision-making and willingness to receive the COVID-19 vaccine in Saudi Arabia (28). Most participants had a high perceived risk exposure and perceived severity which made them more likely to receive the COVID-19 vaccine (28). Participants also believed that the chances of infection would be reduced after vaccination (28). The results of this study are consistent with a Malaysian study that used the HBM to assess willingness to receive the COVID-19 vaccine (29). Perceived susceptibility, perceived severity, perceived benefits, perceived barriers, and cues to action were predictors of intent to receive the COVID-19 vaccine among residents of Malaysia (29). A study conducted in Hong Kong reported that COVID-19 vaccine acceptance was significantly correlated with perceived severity, perceived benefits, perceived barriers, cues to action, self-reported health outcomes, and trust in the healthcare system or vaccine manufacturers (30). Participants who report higher levels of perceived benefits, perceived severity, and cues to action are more willing to get vaccinated (25). Individuals who perceive that a disease will be harmful are more likely to make decisions that protect their health (19). These studies in addition to the current one reflect the individual perceptions and behavioral modifications that will help incline people toward vaccination.

The COVID-19 vaccination decision of unvaccinated participants was reported at a moderate level (53.2%). Factors influencing the decision to receive vaccines in this study included fear of injection, fear of the short and long-term side effects of vaccination, and hesitancy about the efficacy of the vaccine. These results highlighted the importance of addressing issues such as self-preparation for short-term and long-term side effects, including prolonged pain, redness or swelling, at the injection site and trust in the vaccine. The findings are supported by a prior study showing that the COVID-19 vaccine acceptance rate was 41.8% in Thailand (31). A previous study conducted in Bangladesh revealed that 85% of the population had an intention to vaccinate (32). Another study in the United Kingdom and United States showed that 54.1% of respondents would accept a COVID-19 vaccine to protect themselves against the infection (33). Meanwhile, in Hong Kong, 42.2% of respondents to a public telephone survey indicated their intention to receive the COVID-19 vaccine (30). The current study found that underlying disease was inversely associated with the decision to vaccinate against COVID-19. However, when this variable was included in the regression model, it became non-significant. This suggests that a program based on HBM should be developed to encourage COVID-19 vaccination among unvaccinated people in rural areas, particularly those with an underlying disease.

Many unvaccinated participants (39.9%) reported a moderate level of COVID-19 preventive behaviors. These participants were also more likely to wear a medical mask, eat a healthy diet, keep up to date with the pandemic and obtain information about the disease, suggesting that they recognize the benefit of vaccinating against COVID-19. These results suggest that it may be necessary to emphasize the importance of taking preventative action because many participants minimized certain health behaviors such as checking body temperature or avoiding travel to crowded places. This is likely because perceived barriers affected decision-making about health behaviors that prevent infection. Individual protective behavior depends on the presence of the perceived risk, the perceived severity of the disease, and the perceived benefits and barriers to preventing the infection (19). This is also consistent with previous findings that associated the perceived risk of contracting or dying from COVID-19 with better health behaviors such as increased handwashing and avoiding crowds (34). Public health messages that highlight the benefits of health behaviors to prevent COVID-19 infection are likely to be most effective (35). It is also important to focus on removing barriers to COVID-19 vaccination (6).

Health Belief Model responses were found to predict self-care behaviors for COVID-19 prevention (*R*^2^ = 42.5%). The HBM framework helps to predict the causes for individual decisions to adopt preventive measures that will reduce the risk of infection (19, 36). A previous study in Saudi Arabia showed that HBM constructs can be used to assess community protective measures (37). In addition, HBM constructs explained 27% of the variance in COVID-19 preventive behaviors within the general population of Iran (6). Moreover, HBM accounted for 29 and 46% of the variance in behaviors to prevent COVID-19 infection among Iranian adults and adolescents, respectively (38, 39). Previous studies have supported the HBM model for predicting and explaining protective behaviors against infectious diseases such as COVID-19 (40, 41). People use protective behaviors when they feel threatened by a pandemic situation, are aware of the potential risk of the disease and consider the disease serious (perceived severity) (41). At the same time, the information and advice people get from their surroundings or internal environment (guiding the action), the perception toward the benefit (perceived benefit), and (cues to actions) of the behavior, drive them to use preventive behaviors (41, 42). Perceived barriers can weigh the individual decision to get vaccinated against the severity of COVID-19 (41, 42). However, self-efficacy for reducing the threat (the belief that the behaviors will be beneficial) is strongly related to behavior uptake (43). A study of Canadian adults that perceived threat and self-efficacy for preventive behaviors predicted uptake of those behaviors (43). The results of the current study support the HBM theory, which describes perception as a factor that encourages individuals to act to protect themselves against the disease (19). Thus, practicing behaviors that prevent infection could be one of the ways to prevent sickness and death from COVID-19 and other infectious diseases.

This study has some strengths and limitations. A strength is that variables affecting both the possibility of COVID-19 vaccine rejection and hesitancy in the context of the HBM structure were identified. Of these, perceived severity of disease, perceived risk exposure, self-efficacy, perceived benefits and barriers, and cues to action are evident. Responses to these questions can guide the design of vaccination campaigns that target undecided or unvaccinated groups. One limitation to this study is that it was conducted during the third wave of the COVID-19 pandemic. Government agencies have continued public relations and campaigns to get people vaccinated. Moreover, the data was obtained from self-report which can lead to data bias. Another limitation is that the responses of acceptance, refusal, and hesitancy are marked by the temporal context of the pandemic so could change over time. Further studies are needed to examine changes in vaccination intention and infection detection during the COVID-19 pandemic. Because this study was conducted only in upper northern Thailand, and even though it used a probability sampling, these results are not generalizable to the entire Thai population. Larger sample sizes and broader sampling areas are recommended. Demographic factors not included in this study such as religion and tribe should also be considered for next study.

## Conclusion

This study showed that most participants had a moderate level of intention to receive the COVID-19 vaccines. The HBM can be used to predict vaccination intention among unvaccinated people in Thai communities. The findings of the study can be used as a guideline for vaccination policy and community immunization plans. An HBM-based campaign offers an effective framework to resolve beliefs and attitudes for promoting COVID-19 prevention behaviors and vaccination among Thai and other populations. Health information campaigns need to highlight (1) perceived side effects, such as health complications, especially among chronically ill populations, and the efficacy of the vaccines, (2) the benefits of prevention behaviors and advice for overcoming barriers to self-care, and (3) warning signs through social media in order to build trust around making the decision to receive the COVID-19 vaccine, and (4) behaviors the prevent COVID-19 infection. Adding information that is targeted to priority groups, particularly those with chronic illnesses, should also be considered.

## Data Availability Statement

The data supporting the study's findings are available upon request from the corresponding author.

## Ethics Statement

The studies involving human participants were reviewed and approved by University of Phayao Human Ethics Committee, Thailand (UP-HEC 1.2/034/64, approved 18 September 2021). The patients/participants provided their written informed consent to participate in this study.

## Author Contributions

KS and PO-A contributed to the conception and design of the work and contributed revising the work for important intellectual content. TP, SB, NA, PT, and SK contributed the data acquisition. PO-A, MC, and SK contributed the analysis and interpretation of data for the work. KS, TP, and PO-A contributed drafting the work. All authors approved of the final version to be published and agree to be accountable for all aspects of the work in ensuring that questions related to the accuracy or integrity of any part of the work are appropriately investigated and resolved.

## Funding

The authors are grateful to the research project was supported by School of Medicine (MD65-04) and the Thailand science research and innovation fund and the University of Phayao the Unit of Excellence.

## Conflict of Interest

The authors declare that the research was conducted in the absence of any commercial or financial relationships that could be construed as a potential conflict of interest.

## Publisher's Note

All claims expressed in this article are solely those of the authors and do not necessarily represent those of their affiliated organizations, or those of the publisher, the editors and the reviewers. Any product that may be evaluated in this article, or claim that may be made by its manufacturer, is not guaranteed or endorsed by the publisher.
